# Deep-Learning Framework for Efficient Real-Time Speech Enhancement and Dereverberation

**DOI:** 10.3390/s25030630

**Published:** 2025-01-22

**Authors:** Tomer Rosenbaum, Emil Winebrand, Omer Cohen, Israel Cohen

**Affiliations:** 1 Andrew and Erna Viterbi Faculty of Electrical & Computer Engineering, Technion—Israel Institute of Technology, Haifa 3200003, Israel; tomer11r@campus.technion.ac.il; 2 MISTRIX Ltd., Tel Aviv 6492108, Israel; 3 Insoundz Ltd., Tel Aviv 6821231, Israel; emil.winebrand@insoundz.com (E.W.); omer@insoundz.com (O.C.)

**Keywords:** deep filtering, real-time processing, speech dereverberation, speech enhancement

## Abstract

Deep learning has revolutionized speech enhancement, enabling impressive high-quality noise reduction and dereverberation. However, state-of-the-art methods often demand substantial computational resources, hindering their deployment on edge devices and in real-time applications. Computationally efficient approaches like deep filtering and Deep Filter Net offer an attractive alternative by predicting linear filters instead of directly estimating the clean speech. While Deep Filter Net excels in noise reduction, its dereverberation performance remains limited. In this paper, we present a generalized framework for computationally efficient speech enhancement and, based on this framework, identify an inherent constraint within Deep Filter Net that hinders its dereverberation capabilities. We propose an extension to the Deep Filter Net framework designed to overcome this limitation, demonstrating significant improvements in dereverberation performance while maintaining competitive noise-reduction quality. Our experimental results highlight the potential of this enhanced framework for real-time speech enhancement on resource-constrained devices.

## 1. Introduction

Recording speech with distant microphones in rooms often introduces acoustic challenges, most notably background noise and reverberation. These degradations negatively impact the recorded signal quality and hinder the performance of applications such as automatic speech recognition (ASR). The field of speech enhancement aims to address these issues, improving the intelligibility and usability of captured speech. Considerable research efforts have focused on two key areas: dereverberation, which tackles the persistent echoes resulting from sound reflections within the environment, and noise reduction, designed to suppress unwanted sounds and isolate the desired speech signal. Developing practical solutions for these common acoustic distortions remains crucial for achieving robust and reliable performance in real-world applications.

The rise of deep learning and artificial intelligence (AI) has revolutionized speech enhancement, leading to state-of-the-art performance in both noise reduction and dereverberation [1,2,3,4,5]. Recent methods often employ complex neural networks (NNs) architectures trained to directly generate enhanced speech from degraded input signals. This data-driven generative approach can produce remarkably high-quality, even “studio-quality”, speech from challenging acoustic environments. The effectiveness of these techniques is evident in commercially available products like Adobe Podcast [6], showcasing the practical impact of deep learning on enhancing speech clarity and intelligibility.

Despite their effectiveness, these state-of-the-art deep-learning generative methods often rely on large, complex models, leading to relatively slow processing speeds. Furthermore, the reliance on cloud-based processing with expensive hardware to handle the degraded speech input creates a barrier to deployment on edge devices like mobile phones. This reliance on external processing power makes these advanced techniques unsuitable for real-time applications such as communication platforms, video conferencing (e.g., Zoom), and other streaming services where immediate processing is essential.

Traditional signal processing approaches, conversely, rely on mathematical models to describe the signal acquisition and enhancement process. Based on these formulations, solutions are derived, often involving the estimation of model parameters. In many cases, closed-form solutions are attainable only under simplifying assumptions about the signal’s properties, such as assuming a Gaussian distribution. Standard basic methods for noise reduction include the Wiener filter [7], and the minimum variance distortionless response (MVDR) filter [8], along with numerous variants, generalizations, and extensions [9,10]. In speech dereverberation, familiar approaches encompass spectral subtraction [11,12] and inverse filtering techniques [13,14,15,16,17] such as weighted prediction error (WPE) [16,17] and its derivatives [18,19,20,21,22]. While these closed-form solutions offer computational convenience and interpretability, the underlying assumptions often limit their effectiveness and robustness in real-world scenarios with more complex signal characteristics.

A more promising approach for real-world scenarios combines traditional signal processing concepts with the power of deep learning. Rather than directly generating enhanced speech like purely data-driven methods, this hybrid approach formulates a model similar to traditional methods. However, instead of relying on simplifying assumptions, an NN module is trained to estimate the model parameters. This eliminates the need for restrictive assumptions and allows for more robust parameter estimation in complex data distributions. Moreover, this approach often requires only a lightweight NN for effective performance, making it suitable for real-time applications on resource-constrained devices.

Early hybrid approaches to noise reduction focused on predicting masks applied to the observed signal in the short-time Fourier transform (STFT) domain. Instead of deriving closed-form solutions based on signal assumptions, these methods employed lightweight NNs to predict the masks, typically from a window of consecutive STFT frames. While computationally efficient, mask-based approaches exhibited limitations, particularly with challenging transient noises (e.g., a crying baby) containing difficult-to-remove periodic components.

A significant advance came with the introduction of deep filtering [23], where an NN predicts frequency-wise linear complex filters applied directly to the degraded speech in the STFT domain. These deep filtering approaches generally employ filters that can incorporate non-causal future samples, offering potential performance benefits at the cost of increased latency or operating solely on causal past and present information. Furthermore, these filters can leverage information from neighboring frequency bands, a technique known as crossband filtering [24]. This shift towards deep filtering has yielded substantial performance improvements, with some solutions achieving results that are competitive with computationally expensive large-scale generative models while maintaining significantly greater efficiency.

Building on the success of deep filtering, Schröter et al. recently introduced Deep Filter Net [25,26,27], a novel framework distinguished by its exceptionally low computational complexity. This efficiency is achieved through a clever combination of approaches: employing a mask-based approach for the less demanding aspects of speech enhancement and reserving the more computationally intensive deep filtering approach for the more challenging parts. Remarkably, this combined approach achieves state-of-the-art performance comparable to significantly more complex models, even in challenging noise scenarios, offering a compelling balance between enhanced speech quality and computational efficiency.

Recently proposed methods, such as Grouped Temporal Convolutional Recurrent Networks (GTCRN) [28] and FSPEN [29], show significant improvements in terms of computational complexity while maintaining competitive performance in noise reduction compared to Deep Filter Net. Both models rely on applying complex masks to the STFT representation of the input signal. We focus on deep filtering-based methods since dereverberation inherently benefits from approaches utilizing long filters to model late reflections [16]. In this paper, we specifically extend and evaluate Deep Filter Net to address its dereverberation limitations, demonstrating this framework’s potential for real-time speech enhancement in reverberant environments.

While Deep Filter Net demonstrates promising results for real-time noise reduction, its performance in speech dereverberation remains challenging due to its inherent reliance on a short input vector, which is insufficient to effectively model the long temporal dependencies crucial for mitigating late reverberation. As highlighted earlier, effective dereverberation is crucial for robust speech processing in real-world environments. This paper addresses this challenge by improving dereverberation capabilities in existing lightweight frameworks, using Deep Filter Net as a case study. We formulate a general framework encompassing mask-based approaches, deep filtering, and Deep Filter Net as private cases. Through this generalized framework, we identify an inherent constraint within the Deep Filter Net architecture that limits its dereverberation capabilities. Inspired by concepts from traditional signal processing, we propose an extension to overcome this limitation and improve dereverberation performance without compromising its efficient noise reduction.

It is important to note that dereverberation inherently involves modeling long temporal dependencies to address late reflections in reverberant environments. This requirement necessitates using longer filters, which naturally increases computational complexity compared to other tasks like noise reduction. As such, the goal of this work is to demonstrate that significant improvements in dereverberation performance can be achieved with only a modest increase in complexity.

Experimental results validate the effectiveness of our proposed extension, demonstrating significant improvements in dereverberation while maintaining high-quality noise reduction. These findings highlight the potential of this enhanced framework for real-time speech enhancement in reverberant environments and provide a pathway for advancing dereverberation in lightweight models. We share audio samples and results through our project page (See the project page at https://tomermistrix.github.io/deep-filter-net-dereverberation, accessed on 10 December 2024).

The remainder of this paper is organized as follows: Section 2 presents the model and formulates the speech enhancement task. Section 3 overviews previous work and sets the basis for our methods. Section 4 describes our proposed methods. Section 5 details the experimental setup and results. Section 6 concludes this work.

## 2. Model Formulation

Capturing speech signals with a microphone inevitably introduces acoustic distortions from the surrounding environment. A primary source of such degradation is the convolution of the clean speech signal with the room’s acoustic impulse response, resulting in reverberation—the persistence of sound due to reflections within the enclosed space. Additionally, background noise, including any unwanted acoustic signals, complicates acquiring a clear speech recording. This section formalizes the signal model incorporating these degradations and defines the speech enhancement task within this context.

### 2.1. Signal Model

Consider a single speech source represented by the time-domain signal x(n)∈R. Its STFT representation is denoted by $x_{f,t}\in C$, where f=0,⋯,F−1 and t=0,⋯,T−1 represent the frequency and time bins, respectively. The acquired signal y(n), captured by a single microphone, is a combination of the reverberant speech and additive noise:

(1) $$y\left(n\right)=\left(h*x\right)\left(n\right)+v\left(n\right)=\left(h_{e}*x\right)\left(n\right)+\left(h_{l}*x\right)\left(n\right)+v\left(n\right),$$

where h(n) is the room impulse response (RIR), ∗ denotes linear convolution, and v(n) represents the background noise. This work focuses on reverberant environments and transient noise sources, reflecting common real-world acoustic challenges.

A prevalent approach in dereverberation is to decompose the RIR, h(n), into two components based on a predefined delay parameter *D*, typically set to 50 ms. This decomposition separates the early reflections, crucial for speech intelligibility, from the detrimental late reverberation. The first component, $h_{e}\left(n\right)$, comprises the first *D* samples of h(n) and represents the direct sound and early reflections. The second component, $h_{l}\left(n\right)$, consists of the remaining samples and embodies the late reverberation.

Within this framework, speech enhancement aims to design a system *G* that effectively recovers the direct sound and early reflections from the degraded signal y(n) while simultaneously suppressing the background noise and late reverberation. Formally, the desired enhanced signal $\hat{x}\left(n\right)$ is given by:

(2) $$\hat{x}\left(n\right)=G\left(y\left(n\right)\right)\approx \left(h_{e}*x\right)\left(n\right).$$

### 2.2. Traditional Speech Dereverberation

Traditional approaches to speech dereverberation often leverage signal processing techniques to mitigate the effects of late reflections. Among these, inverse filtering is a prominent method that aims to estimate and subtract the reverberant component from the observed signal. This subsection focuses on the inverse filtering approach and its formulation in both the time and STFT domains.

The core concept of inverse filtering involves estimating the coefficients of a linear time-dependent filter, $w\left(n\right)\in R^{M}$. Considering the signal model in (1) and assuming negligible background noise for the present discussion, this filter aims to predict the late reverberation component $\left(h_{l}*x\right)\left(n\right)$ from a delayed version of the observed signal y(n). The direct sound and early reflections are estimated by subtracting the predicted late reflections from the observed signal. Formally, the time-domain inverse filtering approach can be expressed as:

(3) $$y\left(n-D\right)={\left[\begin{array}{ccc} y(n-D) & \cdots & y(n-D-M+1) \end{array}\right]}^{T}\in R^{M},$$

(4) $$G\left(y(n)\right)=y\left(n\right)-w^{T}\left(n\right)y\left(n-D\right).$$

Extending this formulation to the STFT domain is straightforward. Processing in the STFT domain is typically performed frequency-wise, leveraging the convolutive transfer function approximation. The corresponding equations become:

(5) $$y_{f,t-D}={\left[\begin{array}{ccc} y_{f,t-D} & \cdots & y_{f,t-D-M+1} \end{array}\right]}^{T}\in C^{M},$$

(6) $$G\left(y_{f,t}\right)=y_{f,t}-w_{f,t}^{H}y_{f,t-D}.$$

Various inverse filtering methods, such as WPE, employ different algorithms to estimate the filter coefficients w(n) in the time domain or $w_{f,t}$ in the STFT domain. These estimated coefficients are then used in (4) and (6) to perform dereverberation.

### 2.3. Efficient Neural Networks for Speech Enhancement

Targeting real-time applications necessitates computationally efficient speech enhancement solutions. A promising approach combines traditional signal processing principles with the flexibility of deep learning. This hybrid approach leverages a neural network module to estimate parameters for a conventional signal processing method, thereby enhancing performance by refining parameter estimation rather than modifying the underlying process itself. This subsection formalizes this framework.

Given a degraded time-domain signal y(n), the process typically involves computing its STFT representation $y_{f,t}$, applying a parameterized signal processing method, and then transforming the result back to the time domain. For systems operating in the STFT domain, the neural network module, denoted by *F*, usually predicts parameters frequency-wise. However, parameters for all frequency bands are often predicted in a single inference pass.

Let $Y_{t}\in C^{F\times T_{\mathrm{in}}}$ represent a STFT window at time *t*, encompassing all frequency subbands and a window of $T_{\mathrm{in}}$ time frames centered around *t*. This window may, in general, include future frames. Let $F\left(Y_{t}\right)\left(f\right)$ denote the parameters predicted by the neural network for frequency bin *f*. Furthermore, let $y_{f,t}\in C^{F_{w}\times T_{w}}$ represent a localized collection of STFT coefficients centered around $y_{f,t}$, including $T_{w}$ time frames and $F_{w}$ frequency bins. This allows for incorporating temporal context and crossband information (when $F_{w}>1$).

Using these notations, the general framework can be expressed as:

(7) $${\hat{x}}_{f,t}=H\left(y_{f,t},F\left(Y_{t}\right)\left(f\right)\right),$$

where *H* represents the traditional signal processing method parameterized by the neural network’s output *F*. While the entire framework is trained end-to-end to minimize a chosen loss function, only the neural network module *F* has trainable parameters.

For the scope of this paper, we assume $F_{w}=1$, implying no crossband filtering. Additionally, we will maintain a consistent configuration for the input window $Y_{t}$ across all methods discussed below, simplifying the analysis and comparison.

## 3. Related Work

This section reviews established low-complexity speech enhancement methods that serve as the foundation for our proposed approach. These methods, primarily designed for noise reduction, can be viewed as specific cases of the general framework described in (7). While our scope encompasses both noise reduction and dereverberation, we will analyze these existing techniques through the lens of our generalized model.

### 3.1. Ideal Ratio Mask

The Ideal Ratio Mask (IRM) represents a fundamental approach in which a neural network predicts a real-valued mask, $m_{f,t}\in R$, applied directly to the observed STFT coefficients. The enhanced signal is given by:

(8) $${\hat{x}}_{f,t}=m_{f,t}y_{f,t}.$$

This corresponds to a specific case of (7) with the following components:

(9) $$F\left(Y_{t}\right)\left(f\right)=m_{f,t}\in R,$$

(10) $$y_{f,t}=y_{f,t}\in C,$$

(11) $$H\left(y_{f,t},m_{f,t}\right)=m_{f,t}y_{f,t}.$$

While IRM estimation can be computationally efficient using lightweight neural networks, an inherent limitation arises from the real-valued nature of the mask. Since both the observed STFT coefficient $y_{f,t}$ and the enhanced output ${\hat{x}}_{f,t}$ are complex-valued, a real-valued $m_{f,t}$ may not be sufficient to perfectly reconstruct the desired signal, especially in scenarios involving phase distortions.

### 3.2. Complex Ratio Mask

A natural extension of the IRM is the Complex Ratio Mask (CRM), where the neural network predicts a complex-valued mask, $m_{f,t}\in C$, thus addressing the phase limitations of the IRM. In this case, $F\left(Y_{t}\right)\left(f\right)=m_{f,t}\in C$, while $y_{f,t}$ and *H* remain the same as in (10) and (11), respectively.

Although it has addressed the inherent phase limitations of IRM, CRM still faces several other challenges. One notable issue arises from spectral notches in the observed STFT. These notches, often caused by acoustic effects like acoustic early reflections [30], can lead to zero-valued STFT coefficients ($y_{f,t}=0$) even when the corresponding desired signal component is non-zero. In such scenarios, no mask value, real or complex, can perfectly reconstruct the desired signal.

Furthermore, even without spectral notches, training a neural network to accurately predict the complex mask and generalize well across diverse acoustic environments and speakers remains challenging, particularly in significant transient noise. The complex interplay between speech and noise in the STFT domain makes it difficult for the network to learn a robust mapping between the observed signal and the ideal mask.

It is worth noting that in some cases, the terms IRM and CRM are explicitly defined in the literature, such as being derived based on the energy of speech and noise (IRM) [31]. In this paper, we retain the terms IRM and CRM, even though the models are trained to minimize an arbitrary loss function, interpreting them as general descriptors for the respective approaches.

### 3.3. Deep Filtering

Deep filtering represents a significant advancement over CRM, forming the basis for several state-of-the-art real-time speech enhancement methods. Instead of applying a simple gain as in CRM, deep filtering employs a linear combination of multiple STFT coefficients to predict the enhanced signal.

Given a degraded speech signal $y_{f,t}$ in the STFT domain, a neural network, *F*, predicts a time-frequency dependent filter $g_{f,t}\in C^{N}$. Let $g_{f,t,i}$ denote the *i*-th element of $g_{f,t}$. The enhanced signal is then obtained through linear filtering:

(12) $${\hat{x}}_{f,t}=\sum_{i=0}^{N-1}y_{f,t+l-i}g_{f,t,i}^{*},$$

where *l* is the causality factor (with l=0 corresponding to a causal filter) and ${\left(\;\cdot \;\right)}^{*}$ denotes complex conjugation. Note that deep filtering generalizes CRM; setting l=0 and N=1 reduces (12) to the CRM formulation.

Within the framework of (7), deep filtering is characterized by:

(13) $$F\left(Y_{t}\right)\left(f\right)=g_{f,t}\in C^{N},$$

(14) $$y_{f,t}={\left[\begin{array}{ccc} y_{f,t+l} & \cdots & y_{f,t+l-N+1} \end{array}\right]}^{T}\in C^{N},$$

(15) $$H\left(y_{f,t},g_{f,t}\right)=g_{f,t}^{H}y_{f,t},$$

where ${\left(\;\cdot \;\right)}^{T}$ and ${\left(\;\cdot \;\right)}^{H}$ denote the transpose and Hermitian transpose operators, respectively. Deep filtering-based methods typically follow a similar processing pipeline: the degraded speech waveform is transformed to the STFT domain, time-dependent filters are estimated for each frequency band, linear filtering is performed, and the result is transformed back to the time domain to obtain the enhanced speech waveform.

### 3.4. Deep Filter Net

Deep Filter Net [25,26,27] combines deep filtering with IRM to achieve high performance while maintaining a lightweight architecture, primarily focusing on noise reduction. Deep Filter Net recognizes the distinct impact of noise at different frequencies. IRM provides sufficient noise reduction in high frequencies, where noise mainly corrupts the speech envelope. Conversely, deep filtering is employed for more effective noise suppression in low frequencies, where periodic noise components can mix with speech harmonics, especially for transient noises.

**Figure 1:**
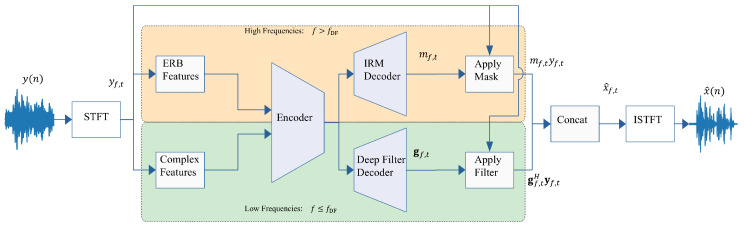
Deep Filter Net framework [27]. High and low frequencies are handled separately.

Figure 1 presents a diagram of the Deep Filter Net. Two separate modules handle high and low frequencies, respectively. Let $f_{\mathrm{DF}}$ be the threshold frequency bin. Frequencies $f>f_{\mathrm{DF}}$ are considered high, while $f\leq f_{\mathrm{DF}}$ are considered low. The high-frequency module operates on equivalent rectangular bandwidth (ERB) features, a downsampled version of the STFT magnitude that enables extremely efficient processing. The predicted IRM in the ERB domain is then interpolated back to the STFT domain and applied to the high frequencies. The low-frequency module operates on the complex STFT representation, but only for $f\leq f_{\mathrm{DF}}$, further reducing computational complexity.

In terms of (7), Deep Filter Net can be formulated as:

(16) $$F\left(Y_{t}\right)\left(f\right)=\left\{\begin{array}{cc} g_{f,t}\in C^{N}, & f\leq f_{\mathrm{DF}},\\ m_{f,t}\in R, & f>f_{\mathrm{DF}}, \end{array}\right.$$

(17) $$y_{f,t}=\left\{\begin{array}{cc} {\left[\begin{array}{ccc} y_{f,t+l} & \cdots & y_{f,t+l-N+1} \end{array}\right]}^{T}\in C^{N}, & f\leq f_{\mathrm{DF}},\\ y_{f,t}\in C, & f>f_{\mathrm{DF}}, \end{array}\right.$$

(18) $$H\left(y_{f,t},F\left(Y_{t}\right)\left(f\right)\right)=\left\{\begin{array}{cc} g_{f,t}^{H}y_{f,t}, & f\leq f_{\mathrm{DF}},\\ m_{f,t}y_{f,t}, & f>f_{\mathrm{DF}}, \end{array}\right.$$

where $g_{f,t}$ and $m_{f,t}$ represent the predicted deep filter and IRM, respectively. Deep Filter Net demonstrates impressive noise-reduction performance, even in challenging environments with transient noises and low signal-to-noise ratios (SNRs), achieving quality comparable to computationally expensive offline models while maintaining a lightweight architecture with approximately 2.5 million trainable parameters in the module *F*. This efficiency makes real-time processing on resource-constrained hardware feasible.

Deep Filter Net’s balance of performance and efficiency makes it a suitable framework for exploring various traditional signal processing concepts. The original authors investigated integrating single-channel Wiener and MVDR filtering [32]. While not guaranteeing the theoretically optimal Wiener or MVDR filters, these adaptations offer a compelling blend of theoretical grounding and practical applicability. For example, in the Wiener filter adaptation, the model predicts a matrix $A_{f,t}\in R^{N\times N}$ and a vector $\phi_{f,t}\in C^{N}$ to construct the filter:

(19) $$g_{f,t}^{\mathrm{wiener}}=\Phi_{f,t}^{-1}\phi_{f,t}\in C^{N},$$

where $\Phi_{f,t}^{-1}=A_{f,t}^{T}A_{f,t}$, ensuring $\Phi_{f,t}^{-1}$ is a positive semi-definite (PSD) matrix. This approach effectively constrains the filter to lie within the set of solutions achievable by multiplying a PSD matrix and a vector—a set that includes the true optimal Wiener filter. Although end-to-end training does not guarantee convergence to the theoretically optimal Wiener filter, this constraint provides a valuable inductive bias. Importantly, the Wiener and MVDR adaptations demonstrated improved performance compared to the original Deep Filter Net, highlighting the potential of incorporating such theoretically informed constraints. This observation motivates further exploration of integrating traditional signal processing concepts within the Deep Filter Net framework, seeking to leverage their theoretical insights for practical performance gains. The next section delves into such extensions, focusing on enhancing dereverberation performance.

## 4. Deep Filter Net for Dereverberation

Although Deep Filter Net is very effective in noise reduction, in terms of dereverberation performance, there is an inherent limitation in the original framework caused by its formulation. In the original framework, e.g., (18), the enhanced signal is predicted from a vector that consists of the last *N* samples of the observed input (assuming causal filter). In contrast, in traditional dereverberation, we predict the late reflections from the previous *M* samples of the delayed version of the input, which is necessary for effective performance. This limitation exists in all approaches presented in the previous section. Furthermore, the number of coefficients required for effective dereverberation is usually larger than that needed for noise reduction, i.e., we assume that M>N.

**Figure 2:**
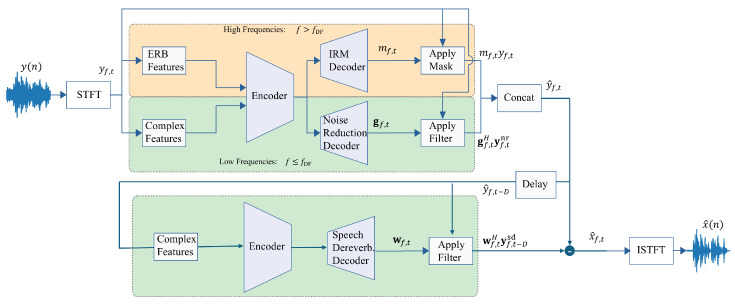
Two-step approach.

**Figure 3:**
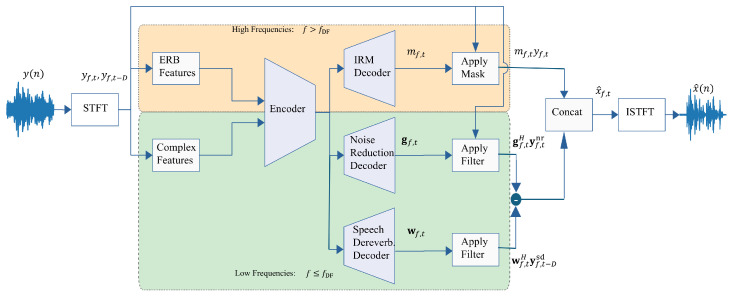
Simultaneous approach.

Our goal is to extend the framework of Deep Filter Net to include concepts of the model in (6) and improve the dereverberation performance without comprising the noise-reduction performance. This extension is not straightforward, and some possible configurations employ the concepts of speech dereverberation and noise reduction. We start with common notations and definitions that will serve as the base of all the approaches we examine. First, we add a new decoder to the original Deep Filter Net architecture, as seen in Figure 2 and Figure 3. We denote the sub-modules in the framework as follows: the encoder *E* predicts the embeddings fed to the 3 decoders. The decoders $D_{\mathrm{IRM}}$, $D_{\mathrm{nr}}$, and $D_{\mathrm{sd}}$ predict the IRM $m_{f,t}\in R$, the noise reduction filter $g_{f,t}\in C^{N}$, and the dereverberation filter $w_{f,t}\in C^{M}$, respectively. Since we have 2 filters of different lengths, we denote the vectors of observed samples as follows:

(20) $$y_{f,t}^{\mathrm{nr}}={\left[\begin{array}{ccc} y_{f,t+l} & \cdots & y_{f,t+l-N+1} \end{array}\right]}^{T}\in C^{N},$$

(21) $$y_{f,t-D}^{\mathrm{sd}}={\left[\begin{array}{ccc} y_{f,t-D} & \cdots & y_{f,t-D-M+1} \end{array}\right]}^{T}\in C^{M}.$$

The proposed method’s primary contribution lies in adding a new decoder and reconfiguring the feedforward flow inspired by traditional dereverberation methods. Unlike the original Deep Filter Net framework, which predicts the enhanced signal based on a limited input window, our approach explicitly incorporates a delayed input vector to model late reflections. This shift mirrors the principles of conventional dereverberation, where late reflections are predicted from delayed input samples. By introducing this mechanism, our method effectively decouples noise reduction and dereverberation processes, enabling more accurate dereverberation without compromising noise-reduction performance. Furthermore, this design leverages insights from traditional signal processing to address the inherent limitations of the original framework, particularly for tasks requiring larger filter lengths for effective dereverberation.

Based on these objects, we explore different design options for the model in (7) according to the following guidelines:

1. Two-step vs. simultaneous speech enhancement: the two-step approach separates the noise reduction and the speech dereverberation. The idea is to reduce background noise first and then attenuate the late reflections from the enhanced speech at the cost of increased latency. Simultaneous processing, on the other hand, can maintain latency that is similar to the original framework, but dereverberation from noisy signals might be more challenging.
2. Low vs. high frequencies: In the original framework, low frequencies are handled using deep filtering, and high frequencies are handled using IRM. Our experiments focus on handling the low frequencies, where most speech energy is located. However, while IRM is adequate for efficient noise reduction in high frequencies, it is unclear how to employ it for dereverberation.

We start by addressing the first point, focusing on the processing of low frequencies.

### 4.1. Two-Step Approach

The two-step approach proposes to enhance the input signal in two steps. In the first step, the flow is the same as the original flow, i.e., the intermediate signal is achieved as in (18). In this step, the new dereverberation decoder is not employed. In the second step, the intermediate signal is fed to the model, but this time, the dereverberation filter is predicted, and the noise-reduction decoder is not employed. The enhanced signal in the second step is achieved as in (6). More specifically, the first step predicts the intermediate signal ${\hat{y}}_{f,t}$ as follows:

(22) $$g_{f,t}=D_{\mathrm{nr}}\left(E\left(Y_{t}\right)\right)\left(f\right),$$

(23) $${\hat{y}}_{f,t}=g_{f,t}^{H}y_{f,t}^{\mathrm{nr}}.$$

Given the intermediate signal with noise reduced, we construct the intermediate window ${\hat{Y}}_{t}$ and the samples vector ${\hat{y}}_{f,t-D}^{\mathrm{sd}}$, and proceed to the second step to remove the late reflections:

(24) $$w_{f,t}=D_{\mathrm{sd}}\left(E\left({\hat{Y}}_{t-D}\right)\right)\left(f\right),$$

(25) $${\hat{x}}_{f,t}={\hat{y}}_{f,t}-w_{f,t}^{H}{\hat{y}}_{f,t-D}^{\mathrm{sd}}.$$

An illustration of the simultaneous approach is presented in Figure 2. This approach might be naive regarding increased latency since the inference consists of 2 feedforwards instead of just one. Still, it provides a complete decoupling between noise reduction and speech dereverberation.

### 4.2. Simultaneous Approach

On the other hand, the simultaneous approach reduces the background noise and attenuates the late reflections in the same feedforward. In this approach, the model predicts the noise-reduction filter and the dereverberation filter from the observed signal simultaneously, contrary to the two-step approach, where the dereverberation filter is predicted from the signal after noise-reduction. The enhanced signal is predicted as follows:

(26) $$g_{f,t}=D_{\mathrm{nr}}\left(E\left(Y_{t}\right)\right)\left(f\right),$$

(27) $$w_{f,t}=D_{\mathrm{sd}}\left(E\left(Y_{t-D}\right)\right)\left(f\right),$$

(28) $${\hat{x}}_{f,t}=g_{f,t}^{H}y_{f,t}^{\mathrm{nr}}-w_{f,t}^{H}y_{f,t-D}^{\mathrm{sd}}.$$

An illustration of the simultaneous approach is presented in Figure 3. This approach is more efficient regarding latency since we predict only one enhanced signal. However, there are two drawbacks to this method. First, the decoder $D_{\mathrm{sd}}$ predicts the dereverberation filter from a noisy input, which is more challenging than predicting it from a noise-free signal. Moreover, since the whole framework is trained in an end-to-end manner, the filters $g_{f,t}$ and $w_{f,t}$ are more coupled, e.g., $w_{f,t}$ might also reduce noise although it was designated for dereverberation and vice versa.

### 4.3. High Frequencies

In both noise reduction and speech dereverberation, since most of the speech energy is in the low frequencies, enhancing the high frequencies is considered more straightforward than the low frequencies. Therefore, we propose that high-frequency enhancement using IRM is also sufficient for dereverberation. Regarding the simultaneous approach, this is straightforward. The enhanced high frequencies, i.e., $f>f_{\mathrm{DF}}$, are obtained according to the original framework as in (18), and the low frequencies are obtained as described in (28). Regarding the two-step approach, in the first step, we obtain the intermediate high frequencies according to the same method, and in the second step, we handle only the low frequencies. We observed that enhancing the high frequencies in the second step also degrades the performance, probably because $D_{\mathrm{IRM}}$ is trained more effectively when the flow consists of only one inference (in this case, the decoder is trained to handle only the observed signal rather than handle both the observed and the intermediate signal). Adding another decoder for the second step is possible, but we find it redundant since we require low-complexity methods.

### 4.4. Computational Complexity

Table 1 compares model efficiency, highlighting the number of parameters and Multiply–Accumulate Operations (MACs) for known lightweight speech enhancement models. Mask-based approaches, such as GTCRN [28] and FSPEN [29], are exceptionally efficient. They operate with only 0.05–0.08 M parameters and 0.03–0.09 G/s MACs, orders of magnitude lower than deep filtering-based methods.

Deep filtering-based approaches typically require more computational resources to achieve enhanced performance. For example, the parameter count in Deep Filter Net increased from 1.8 M in its original version to 2.3 M in Deep Filter Net 3, reflecting the need for additional capacity to improve results. Similarly, our proposed methods (simultaneous and two-step) increase the parameter count to 2.9 M. This reasonable increase is due to adding the second decoder $D_{\mathrm{sd}}$ in the architecture, which is shared by both methods. Since the modules are identical in both the simultaneous and two-step approaches, the number of parameters is the same for both methods. However, the MACs differ between the two methods. The two-step method incurs a higher MAC cost (0.65 G/s) compared to the simultaneous method (0.44 G/s) and Deep Filter Net 3 (0.36 G/s). This is caused by the sequential nature of the two-step approach, which performs two iterations during inference. While this is a limitation of the two-step method, we demonstrate that it significantly improves dereverberation performance, making it a worthwhile trade-off. In future work, we aim to investigate strategies to overcome the sequential nature of the two-step approach and further optimize its computational efficiency.

## 5. Experimental Validation

### 5.1. Implementation Details

To validate the performance of the proposed approaches, we implement the methods upon the official implementation of the latest version of Deep Filter Net [27]. STFT is computed using a 20 ms window with a hop size of 10 ms, and the noise-reduction decoder predicts a 5-tap filter (i.e., N=5). For speech dereverberation, we choose a longer filter and set M=10. The architecture of $D_{\mathrm{sd}}$ differs from $D_{\mathrm{nr}}$ only by the last output layer that is modified to predict the dereverberation filter in the correct dimension.

For training, we use the Deep Noise Suppression (DNS) Challenge dataset that consists of speech, noise, and RIRs sampled at 48 kHz [36]. We adopt the original framework’s loss function, which comprises two components: the spectrogram loss $L_{\mathrm{spec}}$ and the multi-resolution (MR) loss $L_{\mathrm{MR}}$ [25,26]. The spectrogram loss is defined as

(29) $$L_{\mathrm{spec}}\left(x,\hat{x}\right)=\sum_{f,t}\left[{\left|{\left|x_{f,t}\right|}^{c}-{\left|{\hat{x}}_{f,t}\right|}^{c}\right|}^{2}+{\left|{\left|x_{f,t}\right|}^{c}e^{j∠x_{f,t}}-{\left|{\hat{x}}_{f,t}\right|}^{c}e^{j∠{\hat{x}}_{f,t}}\right|}^{2}\right],$$

where ∠a is the phase of the complex number a∈C, and c=0.6 is a compression factor to model the perceived loudness [37]. The intuition of employing two terms in $L_{\mathrm{spec}}$ is related to the architecture of Deep Filter Net, i.e., the first term (magnitude only) guides the IRM decoder while the second term (magnitude and phase) guides the (complex) deep filtering-based decoders. The MR loss is defined as

(30) $$L_{\mathrm{MR}}\left(x,\hat{x}\right)=\sum_{i}\sum_{f,t}\left[{\left|{\left|x_{f,t}^{\left(i\right)}\right|}^{\tilde{c}}-{\left|{\hat{x}}_{f,t}^{\left(i\right)}\right|}^{\tilde{c}}\right|}^{2}+{\left|{\left|x_{f,t}^{\left(i\right)}\right|}^{\tilde{c}}e^{j∠x_{f,t}^{\left(i\right)}}-{\left|{\hat{x}}_{f,t}^{\left(i\right)}\right|}^{\tilde{c}}e^{j∠{\hat{x}}_{f,t}^{\left(i\right)}}\right|}^{2}\right],$$

where $x_{f,t}^{\left(i\right)}$ and ${\hat{x}}_{f,t}^{\left(i\right)}$ are the *i*-th STFT representation with window lengths in {5,10,20,40} ms of the clean and the enhanced signals, respectively, and $\tilde{c}=0.3$. We initialize all modules in the framework except $D_{\mathrm{sd}}$ using the pretrained weights of Deep Filter Net 3 and train according to the following procedure. Initially, we fix the pretrained weights of all modules except $D_{\mathrm{sd}}$ and train only $D_{\mathrm{sd}}$ for the first few epochs. Subsequently, we train all modules together until convergence. We adopted this procedure after observing in our experiments that it outperformed alternative approaches, such as training the entire framework from scratch, in terms of both performance and convergence speed.

For evaluation, we employ the VCTK/DEMAND test set, which was not seen during training, ensuring an unbiased assessment of the proposed methods. To emphasize the generalization of our approach, we evaluate performance across two distinct scenarios: a reverberant condition, isolating the impact of reverberation, and a noisy-reverberant condition, combining both reverberation and additive noise. These two scenarios allow us to demonstrate the effectiveness of our methods in addressing dereverberation both as an isolated challenge and in conjunction with noise, highlighting their robustness in different acoustic environments.

### 5.2. Performance Measures

This study evaluates dereverberation performance using three established metrics frequently employed in the field [38,39]: frequency-weighted segmental SNR (FWSegSNR) [40,41], cepstral distance (CD) [42], and perceptual evaluation of speech quality (PESQ) [43]. It is important to acknowledge the lack of a universally accepted set of objective quality metrics for dereverberation [39]. Consequently, the selected performance measures aim to provide insights into different methods’ relative merits and drawbacks. For a clean reference signal $x_{f,t}$ and its enhanced counterpart ${\hat{x}}_{f,t}$ in the STFT domain, FWSegSNR is calculated as:

(31) $$\mathrm{FWSegSNR}=\frac{1}{T}\sum_{t=0}^{T-1}\frac{\sum_{f=0}^{F-1}w_{f,t}\log_{10}\frac{x_{f,t}^{2}}{{\left(x_{f,t}-{\hat{x}}_{f,t}\right)}^{2}}}{\sum_{f=0}^{F-1}w_{f,t}},$$

where *F* and *T* represent the numbers of frequency bands and time frames, respectively, and $w_{f,t}$ denotes the weight applied to the *f*-th frequency bin at the *t*-th frame. The weights $w_{f,t}$ are determined using standard AI weights [44]. The CD is defined as:

(32) $$\mathrm{CD}=\frac{1}{T}\sum_{t=0}^{T-1}\sqrt{\sum_{m=0}^{M-1}{\left[C_{x}\left(m,t\right)-C_{\hat{x}}\left(m,t\right)\right]}^{2}},$$

where $C_{x}\left(m,t\right)$ represents the cepstral coefficients of the *m*-th Mel band of $x_{f,t}$ [42]. While higher FWSegSNR and PESQ scores signify improved dereverberation, lower CD values indicate better performance. To better illustrate the method’s efficacy, performance gains relative to the observed signal are presented:

(33) $$\Delta \mathrm{FWSegSNR}=\mathrm{FWSegSNR}/{\mathrm{FWSegSNR}}_{\mathrm{observed}},$$

(34) $$\Delta \mathrm{CD}={\mathrm{CD}}_{\mathrm{observed}}/\mathrm{CD},$$

(35) $$\Delta \mathrm{PESQ}=\mathrm{PESQ}/{\mathrm{PESQ}}_{\mathrm{observed}},$$

where ${\left(\;\cdot \;\right)}_{\mathrm{observed}}$ denotes the metric calculated using the observed signal. Higher values consistently represent improved dereverberation performance using this relative metric, with values below 1 indicating performance degradation. We consider these 3 metrics as “dereverberation metrics”.

Further supplementing these established metrics, we incorporate the composite measures for speech enhancement: CSIG, CBAK, and COVL [45]. These metrics offer a broader perspective on enhancement quality, with CSIG quantifying signal distortion, CBAK assessing background noise attenuation, and COVL providing an aggregate score reflecting both distortion and noise reduction. Similarly, instead of presenting the absolute values, we present the gain compared to the score of the observed signal, i.e.,

(36) $$\Delta \mathrm{CSIG}=\mathrm{CSIG}/{\mathrm{CSIG}}_{\mathrm{observed}},$$

(37) $$\Delta \mathrm{CBAK}=\mathrm{CBAK}/{\mathrm{CBAK}}_{\mathrm{observed}},$$

(38) $$\Delta \mathrm{COVL}=\mathrm{COVL}/{\mathrm{COVL}}_{\mathrm{observed}}.$$

These three metrics are considered to be the “noise-reduction metrics”.

In addition to the dereverberation and noise-reduction metrics, we incorporate Real-Time Factor (RTF) as a measure of computational efficiency, which is crucial for assessing the feasibility of real-time processing. RTF is defined as the ratio of the total processing time to the duration of the input signal and is calculated as:

(39) $$\mathrm{RTF}=\frac{T_{\mathrm{processing}}}{T_{\mathrm{input}}},$$

where $T_{\mathrm{processing}}$ represents the time required for the algorithm to process the signal, and $T_{\mathrm{input}}$ denotes the duration of the input signal. Lower RTF values are desirable as they signify higher computational efficiency. The RTF values demonstrate that the proposed methods, while achieving improved performance, still operate within real-time constraints. Note that RTF is strongly dependent on the hardware used for processing. In our scope, all processing times were measured on an Intel^®^ Xeon^®^ CPU @ 2.20 GHz to ensure consistency and relevance for practical deployment.

### 5.3. Results

This section presents the experimental results comparing the proposed approach against two baseline methods, Deep Filter Net 3 [27] and GTCRN [28], using their official implementations. While the available implementation of GTCRN operates at a sampling rate of 16 kHz, both Deep Filter Net 3 and our proposed method perform enhancement at 48 kHz. For consistency in metrics evaluation, the enhanced outputs of Deep Filter Net 3 and our proposed method are subsequently resampled to 16 kHz. We evaluate two variants of our proposed method, differing in their inference approach:

- **Proposed Method (Simultaneous):** This variant performs noise reduction and dereverberation simultaneously within a single inference pass. It utilizes a unified model to process the input and produce the enhanced speech output in one step.
- **Proposed Method (Two-Step):** This variant employs a two-step inference process, as described in Section 4.1.

The performance of each method is assessed on the reverberant and noisy-reverberant VCTK/DEMAND test sets using the metrics described in Section 5.2.

Table 2 summarizes the performance of the different methods on the reverberant test set, while Table 3 presents the results for the noisy-reverberant condition.

#### 5.3.1. Reverberant Condition

Table 2 presents the performance comparison on the reverberant VCTK/DEMAND test set, explicitly isolating the reverberation’s impact without added noise. As anticipated, the proposed two-step method improves the dereverberation-focused metrics: Δ FWSegSNR, Δ CD, and Δ PESQ. It achieves a remarkable 75.3% increase in Δ FWSegSNR relative to the Deep Filter Net 3 baseline, indicating significantly enhanced reverberation suppression. Improvements in Δ CD (2.0%) and Δ PESQ (22.3%) further highlight the perceptual benefits of the two-step approach in reverberant environments. The simultaneous method also shows moderate gains over the baseline in these metrics but performs below the two-step method. The GTCRN baseline, while lightweight and efficient, achieves slightly lower performance than Deep Filter Net 3 in key dereverberation metrics such as Δ FWSegSNR and Δ PESQ. However, it performs comparably in Δ CD and even surpasses other methods in Δ CSIG.

Regarding the noise reduction metrics (Δ CSIG, Δ CBAK, and Δ COVL), all methods exhibit scores close to 1, indicating minimal change in these aspects. GTCRN achieves the highest Δ CSIG, reflecting its strength in signal distortion metrics, while Deep Filter Net 3 performs best in Δ CBAK and Δ COVL. The slight degradation observed in some of these metrics, particularly Δ CSIG for the two-step method, can be attributed to their lesser suitability for evaluating performance in purely reverberant conditions. As these metrics are primarily designed to assess noise-reduction performance, their sensitivity to subtle signal distortions in the absence of noise may not directly indicate dereverberation quality. The key observation remains that the proposed two-step method significantly enhances dereverberation performance, as demonstrated by the substantial gains in the dedicated dereverberation metrics, while maintaining near-unity scores in the noise-reduction metrics, confirming its effectiveness in addressing reverberation without introducing significant noise-related artifacts.

In terms of computational efficiency, the RTF results reveal that all methods achieve low real-time factors, making them suitable for real-time applications. As expected, Deep Filter Net 3 achieves the lowest RTF due to its relatively lower computational complexity, while GTCRN achieves a similarly efficient RTF. The simultaneous method has a slightly higher RTF but benefits from lower latency compared to the two-step approach. Despite the higher RTF for the two-step method, all methods remain computationally efficient, with RTF values well below 0.1. This demonstrates that the proposed approaches while enhancing dereverberation performance, do not compromise real-time processing capabilities.

#### 5.3.2. Noisy-Reverberant Condition

Table 3 presents the performance comparison on the noisy-reverberant VCTK/DEMAND test set, encompassing both reverberation and additive noise. In this more challenging scenario, the proposed two-step method maintains its advantage in key perceptual metrics, achieving the highest Δ PESQ (1.307) and Δ CD (1.118). These results suggest that the focused training of the dereverberation decoder in the two-step approach contributes to improved perceptual quality and reverberation suppression, even in the presence of noise. While the baseline Deep Filter Net 3 retains its edge in Δ FWSegSNR (2.047) and Δ CBAK (2.201), the two-step method remains competitive, demonstrating only a marginal difference in Δ FWSegSNR. Notably, the two-step method now also exhibits the best overall performance, reflected by the highest Δ COVL (1.683). This indicates that the two-step approach achieves the most favorable balance between mitigating signal distortion and suppressing noise and reverberation in this combined degradation scenario. GTCRN, while strong in noise-reduction metrics like Δ CSIG, falls behind in dereverberation metrics such as Δ PESQ and Δ CD. These results reaffirm that GTCRN’s architecture, which focuses on complex masking, is well-suited for noise reduction but may not fully address the long temporal dependencies required for effective dereverberation. While the simultaneous method shows a modest improvement in Δ COVL compared to the reverberant-only condition, it generally performs slightly below the two-step method across the various metrics. These findings further underscore the effectiveness of the proposed two-step training strategy in complex acoustic environments where both noise and reverberation are present.

The RTF results for the noisy-reverberant condition are consistent with those observed in the reverberant condition, further confirming the computational efficiency of all methods. Despite variations in the input conditions, all approaches maintain low RTF values, ensuring suitability for real-time speech enhancement applications.

## 6. Conclusions

This paper addressed real-time speech dereverberation by identifying a limitation in the Deep Filter Net architecture related to its handling of reverberation. We introduced a novel extension inspired by traditional signal processing principles, incorporating a delayed input vector to explicitly model late reflections. Our approach decouples noise reduction and dereverberation processes by reconfiguring the feedforward flow, significantly enhancing dereverberation performance without compromising noise-reduction capabilities. We proposed two inference strategies—simultaneous and two-step—that address this limitation, with the two-step method demonstrating particularly strong improvements in dereverberation metrics.

While this study demonstrates the computational efficiency and real-time suitability of the proposed methods through low RTF values and moderate parameter counts, we acknowledge that we have not yet conducted deployment experiments on physical devices. Future work will explore implementation on edge devices, such as mobile platforms or embedded systems, to validate the practical applicability of the methods under real-world constraints. Additionally, the two-step method incurs a higher MAC cost due to its sequential nature, and future work will investigate strategies to overcome this limitation, such as alternative architectures or optimized processing pipelines.

Beyond these optimizations, the insights gained from our generalized framework and proposed extensions pave the way for advancements in lightweight speech enhancement. The framework’s flexibility enables the integration of traditional signal processing concepts with deep learning, which can be extended to address other challenges, such as source separation, acoustic echo cancellation, and joint optimization of multiple enhancement objectives. By leveraging these possibilities, this work provides a strong foundation for developing robust and efficient solutions for diverse acoustic environments and real-world applications.

## Figures and Tables

**Table 1:** Comparison of model efficiency: Number of parameters and MACs for different models.

Model	Parameters (M)	MACs (G/s)
PercepNet [33]	8	0.80
DCCRN [34]	3.7	14.36
RNNoise [35]	0.06	0.04
GTCRN [28]	0.05	0.03
FSPEN [29]	0.08	0.09
Deep Filter Net [25]	1.8	0.35
Deep Filter Net 3 [27]	2.3	0.36
Ours (Simultaneous)	2.9	0.44
Ours (Two-Step)	2.9	0.65

**Table 2:** Performance comparison on the **reverberant** VCTK/DEMAND test set. For all metrics except RTF, values higher than 1 indicate better performance; for RTF, lower values indicate better efficiency. Boldface indicates the best-performing method for each metric.

Metric	Deep Filter Net 3	GTCRN	Ours (Simultaneous)	Ours (Two-Step)
Δ FWSegSNR	1.313	1.285	1.185	**2.302**
Δ CD	1.023	0.954	1.009	**1.044**
Δ PESQ	1.069	0.984	0.959	**1.307**
Δ CSIG	1.019	**1.047**	0.991	0.977
Δ CBAK	**1.099**	1.031	1.081	1.015
Δ COVL	**1.075**	1.032	1.035	1.029
RTF	**0.038**	0.046	0.052	0.083

**Table 3:** Performance comparison on the **noisy-reverberant** VCTK/DEMAND test set. For all metrics except RTF, values higher than 1 indicate better performance; for RTF, lower values indicate better efficiency. Boldface indicates the best-performing method for each metric.

Metric	Deep Filter Net 3	GTCRN	Ours (Simultaneous)	Ours (Two-Step)
Δ FWSegSNR	**2.047**	1.823	1.496	1.975
Δ CD	1.108	1.103	1.079	**1.118**
Δ PESQ	1.211	1.146	1.222	**1.307**
Δ CSIG	1.331	**1.425**	1.296	1.341
Δ CBAK	**2.201**	1.208	1.965	2.086
Δ COVL	1.575	1.313	1.566	**1.683**
RTF	**0.037**	0.045	0.051	0.081

## Data Availability

Publicly available datasets were analyzed in this study. These data can be found here: DNS Challenge dataset—https://github.com/microsoft/DNS-Challenge (accessed on 2 December 2024).
